# Higher placement stocking density is associated with increased antibiotic use and footpad lesions in conventional broiler production

**DOI:** 10.1016/j.psj.2026.108042

**Published:** 2026-10-24

**Authors:** Y. Slegers, M.G.R. Matthijs, J.J. de Wit, M. Hostens, J.A. Stegeman

**Affiliations:** a Farm Animal Health, Department of Population Health Sciences, Faculty of Veterinary Medicine, Utrecht University, PO Box 80.151, Utrecht, 3508 TD, The Netherlands; b Royal GD, PO box 9, Deventer, 7400 AA, The Netherlands; c Department of Animal Science, Cornell University College of Agriculture and Life Sciences, Cornell University, Ithaca, NY, 14853-4801, USA

**Keywords:** Broiler, Stocking density, Antibiotic, Footpad lesion

## Abstract

High stocking densities in broiler production have been linked to reduced air and litter quality, which can negatively affect broiler welfare. This study assessed the association between placement stocking density and three key health and welfare indicators in conventional broilers: antibiotic treatments, mortality, and footpad lesion (FPL) scores. Data from 29,542 Dutch broiler flocks (2017-2021) were analyzed using generalized linear mixed models. Placement stocking density was not associated with antibiotic treatment during the first week of production but was positively associated with the probability of treatment after the first week (odds ratio 1.08; 95% CI [1.05, 1.11]; p < 0.001). No association was observed with mortality until thinning. Higher placement stocking density was also associated with higher FPL scores (score ratio 1.09; 95% CI [1.08, 1.10]; p < 0.001). Excluding farms with poor historic FPL scores provided similar results. Together, these findings suggest that higher placement stocking density may increase the occurrence or transmission of infectious diseases, leading to increased antibiotic use, while also adversely affecting footpad health. Reducing stocking density may therefore improve broiler health and welfare in conventional production systems.

## Introduction

High stocking densities are common in broiler production to maximize production efficiency but are associated with welfare concerns (EFSA AHAW Panel, 2023). European legislation sets limits on stocking density: a baseline maximum of 33 kg/m^2^, which can be increased to 39 kg/m^2^ if environmental parameters are met, and to 42 kg/m^2^ with additional requirements, including a maximum mortality level (Directive 2007/43/EC). In the Netherlands, the annual average footpad lesion (**FPL**) score is an additional criterion for permission to stock at 42 kg/m^2^ (Regulation WJZ/14101260).

In addition to limiting space per bird, high stocking densities can reduce air (Jones et al., 2005) and litter quality (e.g., Guinebretière et al., 2024; López-López et al., 2022; McKeith et al., 2019; van der Eijk et al., 2023). Poor litter quality increases the risk of contact dermatitis, including FPL and hock burn (Shepherd and Fairchild, 2010). Many studies have shown lower FPL and hock burn scores at reduced densities (e.g., Haslam et al., 2007; Kyvsgaard et al., 2013; Tullo et al., 2017; van der Eijk et al., 2023), although others found no association (e.g, Allain et al., 2009; Bassler et al., 2013; Bergeron et al., 2020; Rayner et al., 2020). These inconsistent findings suggest that the relationship between stocking density and footpad health may depend on additional management and housing factors. Indeed, some authors have argued that farm-specific housing conditions may play a more important role than density alone (Dawkins et al., 2004).

Increased stocking densities have also been associated with increased stress responses, impaired gut health (Dai et al., 2022), and higher susceptibility to infection (Dong et al., 2025). For example, stocking density has been identified as a risk factor for necrotic enteritis (Tsiouris et al., 2015). One experimental study observed increased mortality attributable to infectious causes at higher stocking densities (Shynkaruk et al., 2023). If higher stocking densities increase the occurrence or transmission of infectious diseases, they may also contribute to increased antimicrobial use. However, despite its importance for antimicrobial stewardship, evidence on the relationship between stocking density and antibiotic use under commercial production conditions remains scarce. Previous studies did not find an association between stocking density and total mortality (e.g., Bergeron et al., 2020; Dawkins et al., 2004; Guinebretière et al., 2024; McKeith et al., 2019).

Welfare concerns in conventional broiler production systems have driven development of alternative, higher welfare approaches, such as the Better Chicken Commitment (Abeyesinghe et al., 2025) and the Dutch Better Life system (Saatkamp et al., 2019), combining lower stocking densities with slower-growing breeds. For example, the requirements for one star in the Better Life system include a maximum growth rate of 45 g/d, a minimum slaughter age of 56 d, and a maximum stocking density of 25 kg/m^2^ (de Jong et al., 2022). Between 2012 and 2023, Dutch supermarkets also employed their own welfare concepts, with growth rates between 45 and 50 g/d and stocking densities between 30 and 38 kg/m^2^ (Stadig, 2019).

We previously reported that Dutch alternative production systems, including supermarket concepts and the Better Life system, were associated with fewer antibiotic treatments, lower mortality and lower FPL scores compared with conventional systems (Slegers et al., 2024). However, because these production systems simultaneously implement multiple management changes, including the use of slower-growing breeds and lower stocking densities, the independent contribution of stocking density could not be determined. To address this knowledge gap, the present study therefore focuses exclusively on conventional broiler production, in which breed type is largely uniform with 89% of flocks consisting of Ross broilers (Slegers et al., 2024), to assess the association between placement stocking density and antibiotic treatments, mortality, and FPL scores under commercial field conditions. By evaluating variation in placement stocking density within conventional production systems, the study provides insight into the independent contribution of stocking density to broiler health and welfare.

## Materials and methods

### Data processing

The data used in this study was derived from a dataset on Dutch broiler flocks previously processed for Slegers et al. (2024). In short, flock level data from the Poultry Monitoring Program (PMP) were combined with antibiotic records from the Central Registration of Antibiotics (CRA), which has been used since 2011 by the Netherlands Veterinary Medicines Authority (SDa) for monitoring and benchmarking of all broiler farms and veterinarians in the Netherlands (Bos et al., 2015). Additional information, including FPL scores, total mortality, and floor area (usable space) of the broiler house, was extracted from the Flock Information system Poultry (KIP). Flocks were excluded if they were raised in Patio^TM^ systems (Vencomatic Group, Eersel, the Netherlands), flock size exceeded 80,000 birds, mortality exceeded 15%, they were thinned more than twice, or they had multiple slaughter transports within the last three days. Thinning was defined as the slaughter of part of the flock more than three days before final slaughter.

Only conventional flocks from 2017 to 2021 were included. Flocks were classified as conventional if they were of a fast-growing breed (indicated as such in the dataset) and were slaughtered at a maximum age of 46 days. A total of 31,385 conventional flocks with known floor area were available. Additional exclusion criteria included a slaughter age below 35 days (1^st^ percentile) or thinning age below 20 days, and a placement or final stocking density outside the 1st-99th percentile range. Placement stocking density was calculated from the total number of birds slaughtered and total mortality, divided by floor area. Placement stocking density was selected as the exposure variable because it is determined at the start of the production cycle and is therefore unaffected by subsequent flock performance. Final stocking density was calculated as the weight (in kg) of batches slaughtered at the end date (i.e., excluding thinning) divided by floor area. After excluding flocks with missing veterinary practice ID, 29,542 flocks remained. Fig. 1 describes the data cleaning steps, including the analysis-specific filter steps described in the following paragraph.

**Fig. 1:**
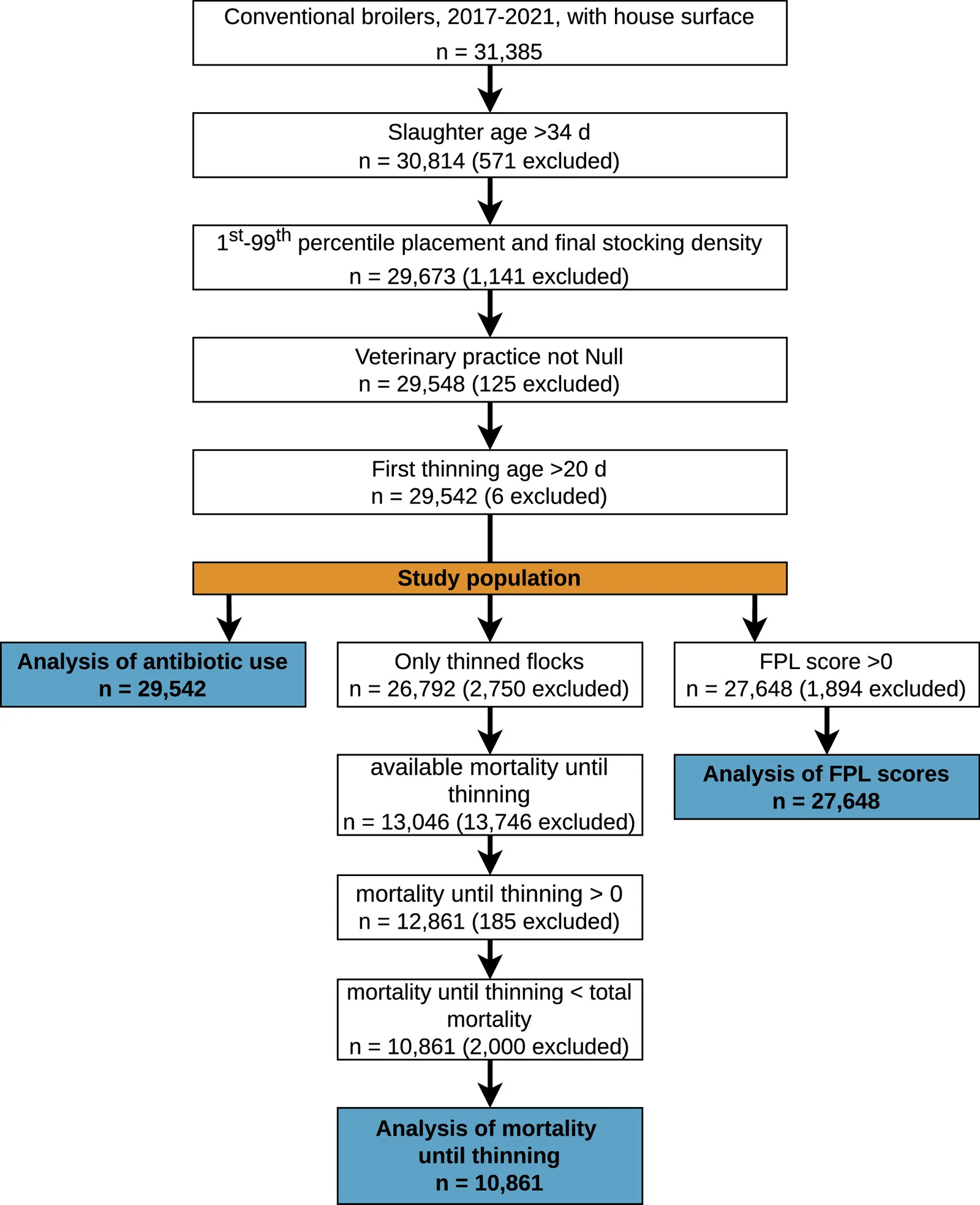
Overview of the data cleaning steps performed for each analysis. The initial dataset was data processed for Slegers et al. (2024).

Outcome variables of interest were antibiotic treatments, mortality, and FPL scores. Antibiotic treatment was included as a binary variable indicating whether a flock received at least one antibiotic treatment, distinguishing treatments administered during the first week from those administered after the first week of the production cycle. To analyze mortality, mortality until thinning was used. End mortality was not used because this measure, calculated by the farmer as total birds found dead and culled divided by placement number, is influenced by the number of thinned birds and thinning age. Analysis of mortality was therefore restricted to flocks with available mortality until thinning, which consequently excluded non-thinned flocks, and further excluded flocks where mortality at thinning was registered as either 0 or equal to or exceeding end mortality, which were likely recording errors. This left 10,861 flocks for analysis of mortality. Analysis of FPL scores was based on scores from the largest slaughtered batch. Flocks with FPL scores of 0 (best score) out of 200 were removed because these values were considered unreliable based on the observed score distribution and likely reflected recording artefacts rather than true absence of lesions (see Slegers et al., 2025a). Analysis of FPL scores were performed using 27,648 flocks.

### Data analysis

Mixed-effects logistic regression models were fitted to estimate the effect of placement stocking density on antibiotic treatments, while mixed-effects negative binomial regression was applied to analyze mortality and FPL scores. The main fixed effect was placement stocking density. Random intercepts for farm and veterinary practice were included in all models. Multiple flocks from the same house were included in the analyses; however, models with random farm and house effects failed to converge due to near-zero variance and where therefore not used. Additional fixed effects (i.e., conditioning variables) were selected a priori using a directional acyclic graph (**DAG**) to identify potential confounders (see the Supplementary materials). First-week antibiotic treatments analysis was controlled for hatch year and quarter. Analysis of mortality until thinning was additionally controlled for age at (first) thinning by including this variable as both offset, to account for differences in time at risk before thinning, and as fixed effect, to capture additional changes in mortality with age. Antibiotics after the first week and FPL scores were controlled for hatch year and quarter, number of thinning events, fraction thinned, and slaughter age. Because thinning strategy is determined during production planning and is related to the intended placement stocking density, number of thinning events and fraction thinned were included as conditioning variables in accordance with the prespecified DAG. Previous FPL scores were an additional possible confounder, since Dutch legislation requires farms with annual house-level FPL scores above 80 to implement improvement measures (Rijksdienst voor Ondernemend Nederland, 2021). Because inclusion of previous annual FPL scores removes 7.157 flocks (24.2%) from analyses, separate models were created with and without this variable.

The relationship between placement stocking density and each outcome was visually assessed for linearity on the model link scale (logit for logistic regression; log for negative binomial regression). As no non-linear patterns were identified, no higher-order terms were included. Collinearity of the final models was assessed using variance inflation factors, with values below 5 considered acceptable. Model results are presented as Odds Ratios (**OR**) (logistic regression) or Rate Ratios (**RR**) (negative binomial regression) with accompanying Wald 95% confidence interval.

All analyses were performed in R version 4.2.2 (R Core Team, 2022) using the following packages: lme4 (Bates et al., 2015), glmmTMB (Brooks et al., 2017), and performance (Lüdecke et al., 2021). The code script for all analyses can be found at https://github.com/decide-project-eu/Slegers-et-al-2026.

## Results

The dataset included 29,542 flocks from 1,654 poultry houses on 499 farms. Median placement stocking density was 21.1 birds/m^2^ (IQR 19.9-22.3 birds/m^2^, range 10.0-25.2 birds/m^2^). Most flocks (90.7%) underwent one or two thinning events, while 9.3% were not thinned (Table 1). Additional descriptive statistics are provided in Table 1, and Fig. 2 shows the distribution of placement stocking density by number of thinning events.

**Table 1:** Description of flocks included in the study.

		Number of thinning events			Total	
		0	1	2		
*n flocks*		2,750 (9.3%)	18,608 (63.0%)	8,184 (27.7%)		29,542
*Placement stocking density (birds/m^2^)*	*Median (IQR)*	16.6 (15.6-18.3)	20.9 (19.9-21.9)	22.6 (21.4-23.6)		21.1 (19.9-22.3)
*Final stocking density (kg/m^2^)*	*Median (IQR)*	39.5 (37.4-41.1)	39.2 (37.2-40.8)	39.0 (37.2-40.6)		39.2 (37.2-40.8)
*Fraction thinned*	*Mean (sd)*	0.0 (0.0)	0.26 (0.06)	0.35 (0.06)		0.26 (0.11)
*First thinning age (d)*	*median (IQR)*	-	34 (32-35)	32 (31-33)		-
*Second thinning age (d)*	*median (IQR)*	-	-	36 (35-37)		-
*Final slaughter age (d)*	*median (IQR)*	39 (36-41)	42 (41-43)	42 (41-43)		42 (41-43)
*Slaughter weight*^1^*(kg)*	*Mean (sd)*	2.39 (0.28)	2.62 (0.22)	2.73 (0.21)		2.63 (0.24)

1 Weight of batch slaughtered at end date

**Fig. 2:**
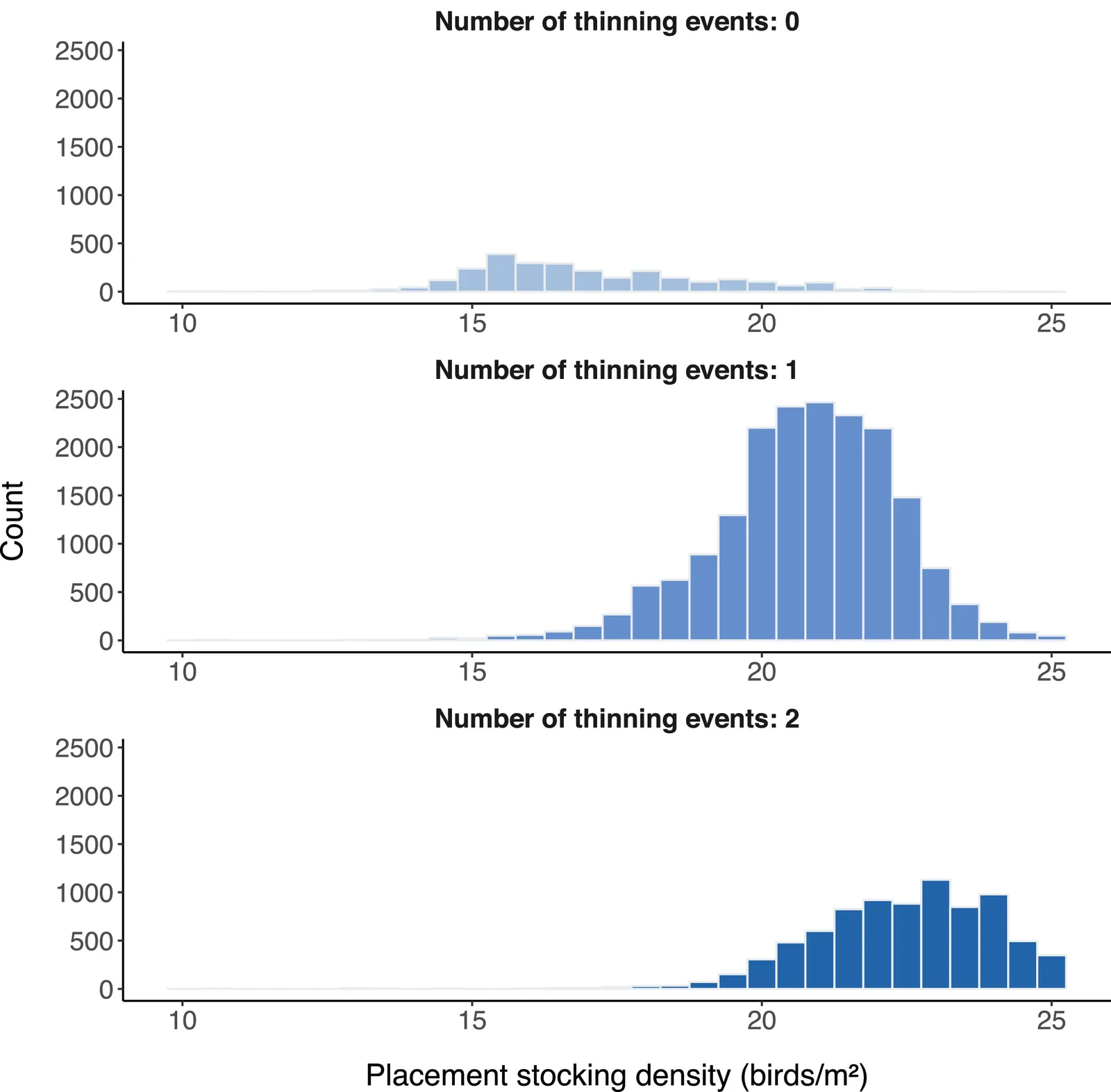
Distribution of placement stocking density by number of thinning events.

Placement stocking density was not associated with antibiotic treatment probability in the first week of the production cycle **(**OR 1.01; 95% CI [0.99, 1.04]; p = 0.31**)**. In contrast, after the first week, higher placement stocking density was associated with an increased probability of antibiotic treatment (OR 1.08; 95% CI [1.05-1.11]; p < 0.001; Table 2). Placement stocking density was not associated with mortality until thinning (RR 1.00; 95% CI [0.99, 1.00]; p = 0.43; Table 3), but was positively associated with FPL scores (FPL ratio 1.09; 95% CI [1.08, 1.10]; p < 0.001, Table 4). The predicted relationships between placement stocking density and the probability of antibiotic treatment after the first week and FPL scores are visualized in Fig. 3. Random effect standard deviations are provided in the Supplementary files (Table S1).

**Table 2:** Odds ratios (OR) for antibiotic treatment in the first week and after the first week. Placement stocking density is the primary variable of interest; other variables were included as conditioning variables and their estimates should not be interpreted causally.

Factor	Level	Antibiotics week 1		Antibiotics after week 1	
		All flocks	Previous year FPL score ≤ 80	All flocks	Previous year FPL score ≤ 80
		OR (95% CI)	OR (95% CI)	OR (95% CI)	OR (95% CI)
Placement stocking density (birds/m^2^)		1.01 (0.99-1.04)	1.02 (0.99-1.05)	1.08 (1.05-1.11)	1.07 (1.04-1.11)
Fraction thinned (scaled)^a^		-	-	0.91 (0.84-0.99)	0.93 (0.85-1.02)
Number of thinning events	0 1 2	-	-	*Ref* 0.95 (0.77-1.17) 0.77 (0.60-1.00)	*Ref* 0.91 (0.71-1.14) 0.77 (0.58-1.03)
Slaughter age (scaled)^a^		-	-	1.07 (1.02-1.12)	1.07 (1.01-1.13)
Hatch year	2017 2018 2019 2020 2021	*Ref* 1.05 (0.95-1.17) 1.00 (0.90-1.12) 1.14 (1.02-1.28) 1.24 (1.08-1.42)	*Ref* 1.13 (0.99-1.29) 1.03 (0.91-1.18) 1.11 (0.97-1.27) 1.22 (1.05-1.42)	*Ref* 1.14 (1.05-1.25) 1.11 (1.01-1.21) 1.01 (0.92-1.11) 0.86 (0.77-0.96)	*Ref* 1.13 (1.03-1.25) 1.07 (0.97-1.19) 0.94 (0.85-1.05) 0.83 (0.73-0.94)
Hatch quarter	1 2 3 4	*Ref* 0.89 (0.81-0.98) 0.83 (0.75-0.91) 1.00 (0.90-1.11)	*Ref* 0.85 (0.76-0.94) 0.81 (0.72-0.90) 0.83 (0.72-0.95)	*Ref* 0.94 (0.87-1.01) 0.86 (0.79-0.93) 0.92 (0.85-1.00)	*Ref* 0.90 (0.83-0.98) 0.82 (0.75-0.90) 0.84 (0.75-0.93)

a The OR of scaled variables represents the change in odds associated with an increase of 1 SD in the explanatory variable. SD of fraction thinned: 0.11. SD of slaughter age: 2.06 d in all flocks, 2.08 in flocks selected on previous year FPL scores.

**Table 3:** Rate ratios (RR) for mortality until thinning. RR represents the ratio of mortality rates, where mortality rate is defined as the number of dead birds divided by thinning age. Placement stocking density is the primary variable of interest; other variables were included as conditioning variables and their estimates should not be interpreted causally.

Factor	Level	All flocks	Previous year FPL score ≤ 80
		RR (95% CI)	RR (95% CI)
Placement stocking density (birds/m^2^)		1.00 (0.99-1.00)	1.00 (0.99-1.01)
Thinning age (d)		1.01 (1.01-1.02)	1.02 (1.01-1.02)
Hatch year	2017 2018 2019 2020 2021	*Ref* 1.02 (1.00-1.04) 0.96 (0.94-0.98) 0.95 (0.93-0.98) 0.98 (0.95-1.01)	*Ref* 1.05 (1.02-1.08) 0.97 (0.95-1.00) 0.96 (0.93-0.98) 1.00 (0.96-1.03)
Hatch quarter	1 2 3 4	*Ref* 0.98 (0.96-1.00) 0.94 (0.92-0.96) 0.95 (0.93-0.97)	*Ref* 0.97 (0.95-1.00) 0.94 (0.92-0.97) 0.92 (0.90-0.95)

**Table 4:** Effect sizes for footpad lesions (FPL). FPL score ratio is the ratio of FPL scores between levels of each explanatory variable. Placement stocking density is the primary variable of interest; other variables were included as conditioning variables and their estimates should not be interpreted causally.

Factor	Level	All flocks	Previous year FPL score ≤ 80
		FPL score ratio (95% CI)	FPL score ratio (95% CI)
Placement stocking density (birds/m^2^)		1.09 (1.08-1.10)	1.09 (1.08-1.10)
Fraction thinned (scaled)^a^		0.96 (0.93-0.98)	0.96 (0.93-0.99)
Number of thinning events	0 1 2	*Ref* 1.15 (1.07-1.24) 1.18 (1.08-1.29)	*Ref* 1.13 (1.04-1.23) 1.17 (1.06-1.30)
Slaughter age (scaled)^a^		1.10 (1.08-1.12)	1.10 (1.08-1.12)
Hatch year	2017 2018 2019 2020 2021	*Ref* 0.92 (0.89-0.95) 1.06 (1.02-1.09) 0.86 (0.83-0.89) 0.67 (0.64-0.70)	*Ref* 0.87 (0.84-0.90) 1.06 (1.02-1.10) 0.86 (0.83-0.89) 0.65 (0.62-0.68)
Hatch quarter	1 2 3 4	*Ref* 0.75 (0.73-0.77) 0.90 (0.88-0.93) 1.10 (1.07-1.13)	*Ref* 0.77 (0.75-0.80) 0.93 (0.90-0.96) 1.16 (1.11-1.20)

a The FPL score ratio of scaled variables indicates the rate of increase in FPL associated with an increase of 1 SD in the explanatory variable. SD of fraction thinned: 0.11. Slaughter age SD: 2.06 d in all flocks, 2.08 in flocks selected on previous year FPL scores.

**Fig. 3:**
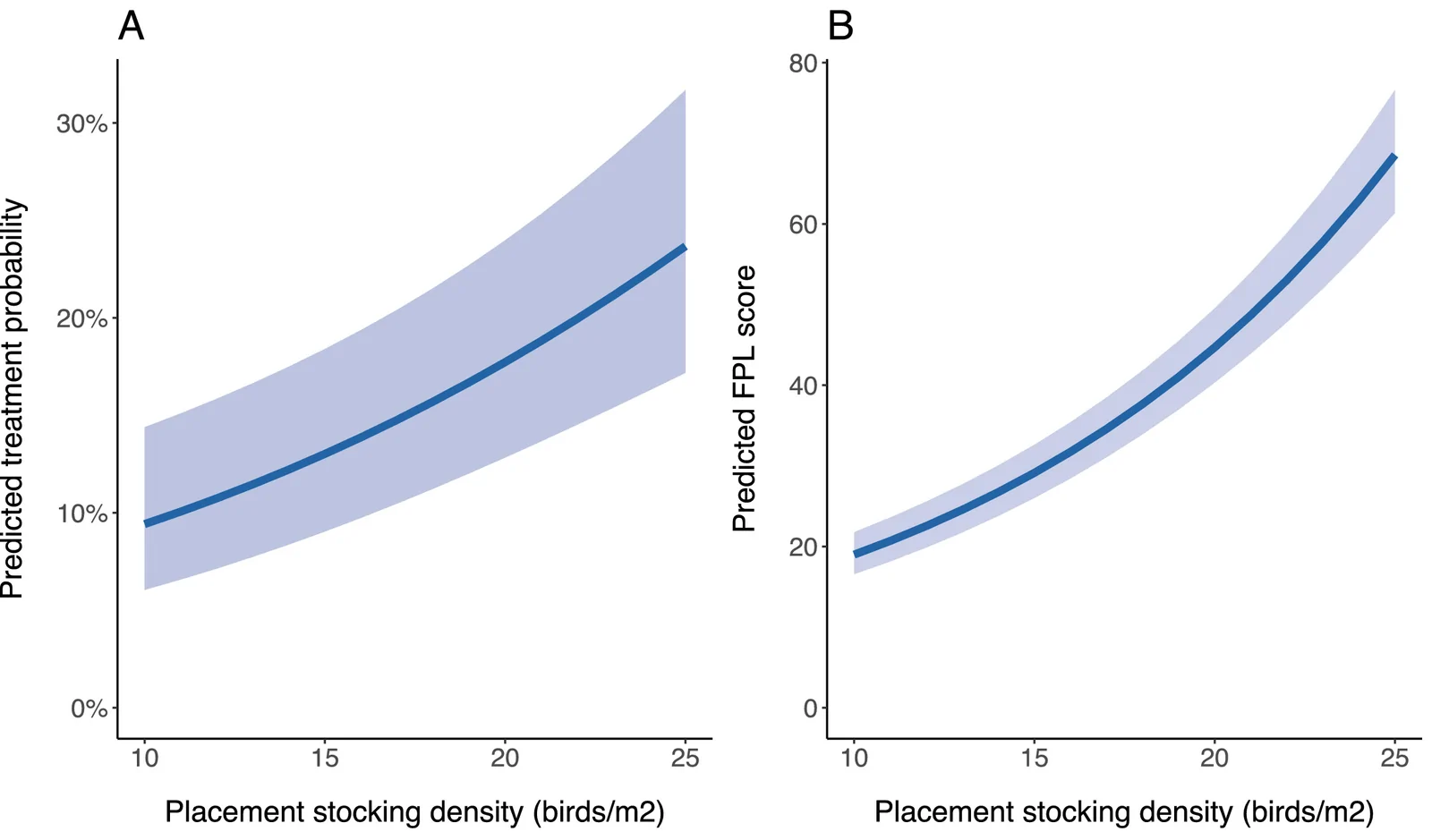
Predicted outcomes (marginal means) plotted against placement stocking density. A) Antibiotic treatment after week 1; B) Footpad lesion (FPL) scores. The shaded area indicates the 95% CI.

## Discussion

This study investigated the association between placement stocking density and antibiotic treatments, mortality, and FPL scores, three key health and welfare indicators, in conventional fast-growing broilers. The observational study design enabled the analysis of a large data set comprising 29,542 flocks reared under commercial farm conditions, where management practices, including ventilation, litter management, and placement of feeders and drinkers, reflected actual farm practices. Consequently, the results are likely to be generalizable to commercial broiler production in the Netherlands and other countries with similar production systems.

### Relationship between placement stocking density and antibiotic treatment

Placement stocking density was not associated with antibiotic treatment probability during the first week of the production cycle. This was expected, because stocking density in kg/m^2^ remains relatively low during the first week because the chicks are still small. As the production cycle progresses, however, bird size increases, reducing the available space per bird and potentially compromising air and litter quality (Dawkins et al., 2004; Tullo et al., 2017; Van der Eijk et al., 2023). Consistent with this, higher placement stocking density was associated with a higher antibiotic treatment probability after the first week of production. A plausible explanation is that the deterioration in environmental conditions associated with higher stocking densities increases susceptibility to infections (Dong et al., 2025), while closer contact between birds may facilitate pathogen transmission within the flock. Together, these mechanisms could increase the likelihood that antibiotic treatment becomes necessary. It should be noted, however, that antibiotic treatment is an indirect measure of infectious disease occurrence. Treatment decisions may also be influenced by factors such as farmer and veterinarian decision-making, treatment thresholds, and prescribing practices. Nevertheless, the need for antibiotic treatment generally reflects the occurrence of clinically relevant disease under commercial conditions, making antibiotic use a meaningful indicator of flock health from both an animal health and antimicrobial stewardship perspective.

To our knowledge, only one previous study has specifically investigated the relationship between stocking density and antibiotic use under commercial conditions (Tarakdjian et al., 2020). In contrast to our findings, that study found no difference in antibiotic use between broiler farms with high and low stocking densities. However, the farms operating at lower stocking densities did so because of restrictions due to inadequate air quality, introducing an important source of confounding that complicates comparison with the present study.

### Relationship between placement stocking density and footpad lesions

FPL scores increased with higher placement stocking density. This finding is biologically plausible, as higher stocking densities are associated with poorer litter quality (Dawkins et al., 2004; Tullo et al., 2017; van der Eijk et al., 2023), while wet litter is a well-established risk factor for the development of FPL (de Jong et al., 2014; Taira et al., 2014). Our findings add to a body of evidence indicating that higher stocking densities adversely affect footpad health. Several observational field studies (Haslam et al., 2007; Kyvsgaard et al., 2013; Tullo et al., 2017) and experimental studies (Dozier et al., 2006; Mocz et al., 2022; Guinebretière et al., 2024) similarly reported increased FPL at higher stocking densities. However, other field studies (Allain et al., 2009; Bassler et al., 2013; Bergeron et al., 2020) and on-farm experimental studies (Dawkins et al., 2004; Rayner et al., 2020) did not detect such an association. These apparently conflicting findings may partly reflect differences in the range of stocking densities studied, but also differences in housing conditions and management practices that influence litter quality independently of stocking density, such as ventilation and litter management. In addition, variation in FPL scoring methods and statistical approaches may have contributed to the inconsistent results. Collectively, the available evidence suggests that stocking density is an important determinant of footpad health, although its impact is likely to depend on the broader production environment in which broilers are kept.

### Relationship between placement stocking density and mortality

No association between placement stocking density and mortality until thinning was found. This is in line with previous studies, under both commercial (Dawkins et al., 2004; McKeith et al., 2019; Bergeron et al., 2020) and experimental conditions (e.g., Astaneh et al., 2018; Buijs et al., 2009; Guinebretière et al., 2024; Shynkaruk et al., 2023), which generally reported no association between stocking density and total mortality. Nevertheless, Shynkaruk et al. (2023) observed higher mortality due to infectious causes in broilers stocked at 41.5 kg/m^2^ than at 31 kg/m^2^ between 21 and 34 days of age. However, infectious causes only accounted for 11% of total mortality during this period. Similarly, first-week mortality, which accounts for almost a third of total mortality (Forseth et al., 2024; Slegers et al., 2025b), is largely determined by chick quality (Yerpes et al., 2020) and appears unrelated to stocking density (Slegers et al., 2025b). Together, these findings suggest that any effect of stocking density on infectious mortality may be diluted when overall mortality is considered.

In the present study, only mortality until thinning could be analyzed because total mortality is not a reliable measure in thinned flocks. Consequently, any effect of stocking density during the final period of the production cycle may have been missed. Future studies should therefore use cumulative mortality, which is unaffected by thinning. In addition, first-week mortality could be separated from later mortality.

### Study strengths and limitations

Although observational studies cannot establish causality with certainty, careful study design and analysis can strengthen the interpretation of observed associations. In the present study placement stocking density clearly occurred before the outcomes, reducing the potential for reverse causation, and potential confounders were identified a priori. Nevertheless, as with any observational study, residual confounding cannot be excluded.

First, placement stocking density may be influenced by factors such as hatchery availability, slaughterhouse demand, and seasonal considerations, but may also be reduced following poor production results. Because Dutch legislation requires farms with persistently high annual FPL scores to reduce stocking density (Rijksdienst voor Ondernemend Nederland, 2021), previous FPL scores may also reflect underlying management practices associated with current flock performance. To account for this, analyses were repeated after excluding houses with poor historic FPL scores (more than 80), which did not substantially affect the results.

Second, the analyses assume that thinning strategy is determined during production planning. In Dutch commercial broiler production, the number of thinning events and the approximate fraction of birds removed are generally planned before chick placement and are therefore closely related to the intended placement stocking density. Consequently, these variables were included as conditioning variables in accordance with the prespecified DAG. Although limited adjustments during production cannot be excluded, these are unlikely to have significantly affected the results.

Finally, selection bias cannot be completely excluded. This is unlikely to have affected the analyses of antibiotic use, which included all available nationwide records. However, exclusion of flocks without mortality data, which was missing in a substantial proportion of flocks, or unreliable FPL scores may have introduced bias if missingness was related to flock performance.

### Comparison with alternative production systems

In a previous study, flocks from alternative production systems had fewer antibiotic treatments, lower mortality and lower FPL scores than conventional flocks (Slegers et al., 2024). These systems combine lower stocking densities with other management changes, including the use of slower-growing breeds, making it impossible to distinguish the independent contribution of stocking density. By focusing exclusively on conventional broilers, the current study provides additional insights into the role of stocking density.

Conventional broilers in the present study were placed at densities ranging from 10.0 to 25.2 birds/m^2^ (IQR 19.9-22.3 birds/m^2^), partly overlapping the densities permitted in alternative production systems (Saatkamp et al., 2019). Based on the fitted model, reducing placement stocking density from 21 to 15 birds/m^2^ was associated with an estimated 37% lower odds of antibiotic treatment after the first week and 40% lower FPL scores, assuming all other variables remained equal. These improvements are smaller than those previously observed in alternative production systems, where first-week antibiotic treatment probability and mortality were also substantially lower (Slegers et al., 2024). This suggests that the improved health and welfare observed in alternative production systems cannot be explained by reduced stocking density alone, but also reflect the contribution of other characteristics, such as slower-growing breeds. Nevertheless, the present findings indicate that reducing stocking density in conventional production systems may still lead to a meaningful improvement in antibiotic use and FPL. This is especially relevant in countries where conventional broiler production remains the dominant production system.

### Suggestions for further study

The present study identified clear associations between placement stocking density, antibiotic use and FPL scores. Although the observational design does not permit definitive causal inference, the findings support further investigation of these relationships under controlled commercial conditions. In particular, experimental studies should evaluate the effect of stocking density on infectious disease occurrence using direct measures of disease, such as diagnostic testing or necropsy findings, rather than antibiotic use alone. Evaluating a broader range of stocking densities, including those used in higher-welfare production systems, would further improve understanding of the relationship between stocking density and broiler health and welfare.

## Conclusion

Higher placement stocking densities were associated with increased antibiotic use after the first week and higher FPL scores, whereas no association with mortality until thinning was found. Together with previous literature, these findings seem to support the hypothesis that lowering stocking density can improve broiler health and welfare in conventional broiler production. However, further experimental studies are needed to confirm whether the associations reflect causal effects.

## Declaration of AI and AI-assisted technologies in the writing process

During the preparation of this work the first author used ChatGPT in order to refine language and to aid in creating R plots. After using this tool, the authors reviewed and edited the content as needed and take full responsibility for the content of the publication.

## Author contributions

Yara Slegers conducted literature research, performed the statistical analyses, and wrote the original draft of the manuscript. Arjan Stegeman was responsible for study conception. Mieke Matthijs, Sjaak de Wit and Arjan Stegeman helped to shape the data analyses and interpret the findings. Miel Hostens aided in data processing. All authors were involved in drafting and critically revising the manuscript for its intellectual content, and reviewed and approved the final version submitted for publication. They are accountable for all aspects of the work and will ensure that any questions regarding the accuracy or integrity of any part of the article are appropriately addressed.

## Disclosures

The authors declare that they have no known competing financial interests or personal relationships that could have appeared to influence the work reported in this paper.

## Footnotes

Supplementary material associated with this article can be found, in the online version, at doi:10.1016/j.psj.2026.108042.
