# Supplementary material for: Higher placement stocking density is associated with increased antibiotic use and footpad lesions in conventional broiler production
Source: Poult Sci. 2026 Oct 24;105(12):108042. doi: 10.1016/j.psj.2026.108042 (PMC13735243; doi:10.1016/j.psj.2026.108042)
Supplement: Supplementary file 1 [file mmc1.docx]

**Supplementary files**

*Table S1. Standard deviations (sd) from random farm and veterinary practice effects in all models.*

| **Model** | **Random effect sd** | |
| --- | --- | --- |
|  | **Farm** | **Veterinary practice** |
| Antibiotics in week 1 | 1.15 | 0.60 |
| Antibiotics after week 1 | 0.90 | 0.75 |
| Mortality until thinning | 0.25 | 0.13 |
| Footpad lesion scores | 0.48 | 0.11 |

**Directed acyclic graphs (DAGs)**

These DAGs were created with expert knowledge and were used to identify variables to control in the analyses. Multiple assumptions were made:

1) Farmers plan how many birds are thinned before stocking and adjust placement stocking density accordingly. Scheduled thinning is assumed to be equal to actual thinning.
2) The combination of variables times thinned and fraction thinned are sufficient to measure the effect of thinning.
3) Quarter of the year captures effects of season

For simplicity, mortality is left out of the DAG, except when this is the outcome of interest. In reality, mortality at time t affects stocking density at time t+1, which in turn can affect mortality at time t+2, etc. Likewise, mortality at time t affects antibiotic treatments at time t+1, which affects mortality at time t+2, etc. These effects do not influence the choice of control variables.

Variables in blue are identified as confounders. Variables in italics are unmeasured variables.
